# Wide Variation of Squeezing Force and Dispensing Time Interval among Eyedropper Bottles

**DOI:** 10.1155/2019/7250563

**Published:** 2019-04-16

**Authors:** Kenji Kashiwagi

**Affiliations:** Department of Ophthalmology, Faculty of Medicine, University of Yamanashi, 1110 Shimokato Chuo, Yamanashi, Japan

## Abstract

**Purpose:**

We aimed to investigate squeezing force and dispensing time interval of the first and second eye drops among a variety of eyedropper bottles and to clarify associated factors within these parameters.

**Methods:**

A total of 87 eyedropper bottles were involved in this study. We developed a squeezing force measuring system consisting of a syringe pump, digital force gauge, and custom-made test stand to measure the squeezing force and dispensing interval. The eyedropper bottle was housed in the system vertically, and measurements were repeated five times. We investigated the differences in squeezing force and dispensing interval by categories, including those that targeted ocular disease, brand or generic eyedroppers, shapes of eyedropper bottles, and the presence of a membrane filter inside the tip of the eyedropper bottle.

**Results:**

The mean squeezing forces of the first drop and the second drops were 8.3 ± 3.0 N and 10.4 ± 3.2 N, respectively. Both squeezing forces had a wide variation of more than threefold. A mean interval between the first and second drops was 3.1 ± 1.2 sec with a maximum difference of more than sevenfold. Round shapes of eyedropper bottles and the presence of filter membranes significantly increased the squeezing force.

**Conclusions:**

The squeezing force of eyedropper bottles was very wide, which may affect proper eye drop instillation. Unification of eyedropper bottles or developing mechanical aids may be useful for proper eye drop treatment.

## 1. Introduction

Eye drop instillation is the main treatment for ophthalmic medicine, and the majority of patients with ocular diseases use eye drops for daily ophthalmic care. Eye drop instillation requires drops to be properly placed in the conjunctival sac by the amount of one droplet. However, there have been multiple problems associated with eye drop instillation in actual clinical practice; the eye drop does not enter the conjunctival sac properly, multiple eye drops are dropped into one eye in a single trial, or the bottle tip makes contact with the ocular surface or eyelash, resulting in ocular surface damage or bacterial contamination inside the eyedropper bottle [1–3]. Solomon et al. demonstrated that up to 80% of patients use an incorrect technique [2], and Tatham et al. reported that 54.1% of patients have poor eye drop instillation technique [1]. Improper technique may contribute to excessive medication waste, increasing medical cost, poorer outcomes, decreasing therapeutic efficacy, lower patient satisfaction, and ocular surface injuries [1]. Newman-Casey et al. reported that difficulty with eye drop administration resulted in poor adherence [4].

Some possible reasons for the difficulty of eye drop instillation have been reported [2]. The factors affecting the patient include poor visual function [5], insufficient backbending of the neck during administration, number of medications used, and the complexity of the administration. Moreover, ease of use of the eyedropper bottle could be another important item for correct eye drop instillation. Many patients who require eye drop treatment do not have the necessary squeezing power for proper eye drop administration due to aging or other diseases, such as rheumatism. We have previously reported that glaucoma patients use on average two types of eye drops [6]. Some reports show different squeezing forces among eyedroppers [7–11], which may affect proper usage.

There have been reports of likely influences of proper eye drop instillation caused by eyedroppers, but the past reports have mainly focused on eyedropper bottles for ocular hypotension. Recently, the variations of eyedropper bottles have drastically increased. For instance, many generic eyedropper bottles were introduced, and eyedropper bottles with filter membranes in the tips to eliminate preservatives from eye drop solution were also developed. There are only a limited number of reports in which these eyedropper characteristics were investigated.

In this study, we employed a variety of eye drops used in clinical practice in Japan, including eyedropper bottles with filter membranes in the tips. We investigated characteristics of eyedropper bottles, such as squeezing force for the first and second eye drops, and their dispensing time interval. We also investigated factors associated with these characteristics to facilitate a way to improve eye drop instillation by modifying the eyedropper bottle.

## 2. Methods

This study did not require any ethical actions because no animals or human beings participated in this study.

### 2.1. Investigated Eyedropper Bottles

Among the ophthalmic solutions approved as prescription or ophthalmic test drugs by the Ministry of Health, Labor and Welfare of Japan, those that pharmaceutical companies agreed to provide for this study were subject to investigation. Details of investigated medications are shown in Table 1 and Supplemental Table A.

### 2.2. Squeezing Force Measuring System

**Figure 1:**
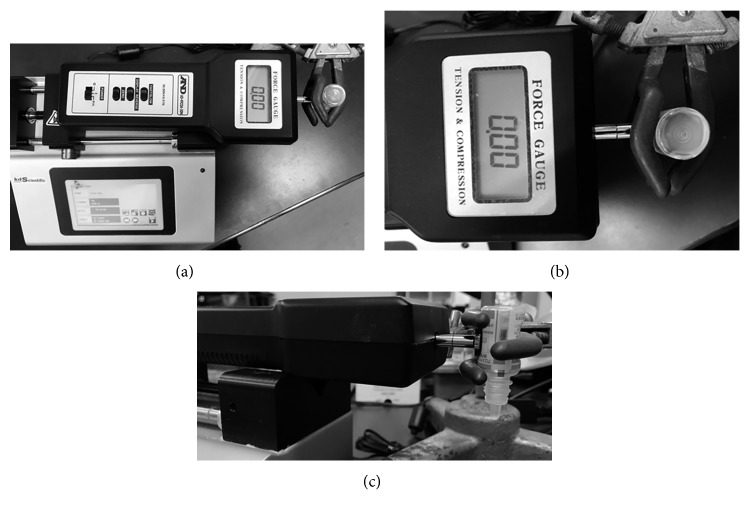
Squeezing force measuring system: (a) overview of the system; (b) magnified vertical image focusing on the eyedropper and compressing tip; (c) magnified side view focusing on eyedropper and compressing tip.

We prepared a squeezing force measuring system, referring to a report by Moore et al. [8]. The system consisted of a syringe pump (Legato 200, KD Scientific), digital force gauge (AD-4932A-50 N, A and D Co. Ltd., Tokyo, Japan), and custom-made test stand. (Figure 1) The syringe pump was designed to apply constant pressure to the eyedropper bottle by mimicking fingertip contact with the eyedropper bottle (Figures 1(b) and 1(c)). The eyedropper bottle was housed in the system vertically, and clamps were adjusted until the compressors were located at midbottle length. The tip of the digital force gauge was then adjusted until its sensor was centered on the eyedropper bottle. Bottles with a square and dimple shape instead of a round shape were compressed at their thinnest dimensions, as this represents the method most likely to be utilized by patients when instilling eye drops.

### 2.3. Measurement of Squeezing Force

All studies were performed at room temperature. Starting at 0 Newton force (N) and 0 millimeters (mm) displacement, the gauge was advanced in 0.5 mm/sec until a drop of ophthalmic solution fell from the bottle. We employed 0.5 mm/sec as a displacement speed because we investigated that this speed was close to that of some patients in the preliminary experiment. Required squeezing forces for the first and second eye drops and an interval of dispensing time between the first and second eye drops (hereafter referred as interval) were recorded. The force gauge was retracted to the neutral position, and any residual eye drop solution at the tip of the bottle was wiped clean after the measurement. The first two measurements were performed as a test. Mean values of the following five measurements were adopted for analysis.

### 2.4. Investigated Parameters and Statistical Analysis

We investigated the required squeezing forces for the first and second eye drops and the time interval between the two drops. As associated parameters to these values, we employed ocular disease category, brand or generic eye drops, shape of the eyedropper bottle, and presence of the membrane filter inside the tip of the eyedropper bottle. The shape of the eyedropper bottle was examined by dividing it into a circular cross section (referred to as the round shape), a central recessed shape (referred to as the dimple shape), and a square shape. Statistical analysis was performed on JMP (SAS Institute Inc., Cary, NC). The Mann–Whitney *U* test or repeated ANOVA were used for comparing force gauge and intervals. The Tukey–Kramer HSD test was used to investigate correlation of the shape of eyedropper bottles, branded or generic eye drops, and intervals with squeezing force. Multivariate regression analysis was employed for investigating associated factors to squeezing force. *P* values with less than 0.05 were considered significant. The values are presented as the means ± standard deviations.

## 3. Results

### 3.1. Investigated Eyedropper Bottles

Table 1 summarizes investigated eye drops by category of ocular disease in this study. The total number of eyedropper bottles was 86; the most common type was for glaucoma, followed by those for dry eye treatment. The shapes of the eyedropper bottle included 53 of the round shape, 21 of the dimple shape, and 12 of the rectangular shape. Details of investigated eye drops are shown in Supplementary Table A. The numbers of branded eyedropper bottles and generic eyedropper bottles were 37 and 49, respectively. It has been reported that preservatives exert several adverse effects on the ocular surface [12–14], even visual function [15]. Some eyedropper bottles attach a filter membrane at the tip to eliminate preservatives from the eye drops. Of all employed eyedropper bottles, a total of eight eyedropper bottles installed a filter membrane in their tip in this study. Since we noticed a large difference in squeezing force between eyedropper bottles with and without a filter membrane in a preliminary experiment, we separately investigated eyedropper bottles without filter membranes from those with filter membranes.

### 3.2. Squeezing Force and Intervals among Eyedropper Bottles without Filter Membranes

Table 2 shows squeezing force of 78 eyedropper bottles without filter membranes. Mean squeezing force of the first drop was 8.3 ± 3.0 N, ranging from 4.2 N to 15.7 N. The ratio between the maximum and the minimum squeezing force was 3.7 times. Mean squeezing force of the second drop was 10.4 ± 3.2 N, ranging from 5.6 N to 18.1 N, which is significantly greater than that of the first drop (*P* < 0.0001). The ratio between the maximum and the minimum squeezing force was 3.3-fold. A mean interval between two drops was 3.1 ± 1.2 sec, ranging from 7.3 sec to 1.0 sec. The ratio between the longest and the shortest interval was 7.3-fold. A mean difference in squeezing force between the first and second drops was 2.0 ± 0.9 N, ranging from 0.2 N to 4.8 N. The ratio between the maximum and the minimum difference in squeezing force was 20.7-fold.

### 3.3. Associated Factors with Squeezing Force

The shape of the eyedropper bottles was significantly associated with squeezing force of the first drop. The round-shaped eyedroppers required a significantly greater squeezing force than the square-shaped and dimple-shaped eyedroppers. In contrast, the shape of eyedropper bottles did not show a significant association with the squeezing force of the second drop (Table 3). Round-shaped eyedropper bottles showed the shortest interval followed by dimple-shaped eyedropper bottles, and there were significant differences between round shapes and square shapes and round shapes and dimple shapes (Table 4). No parameters show any significant difference between branded eye drops and generic eye drops.

### 3.4. Comparison of Squeezing Force in Each Category of Ocular Disease

**Figure 2:**
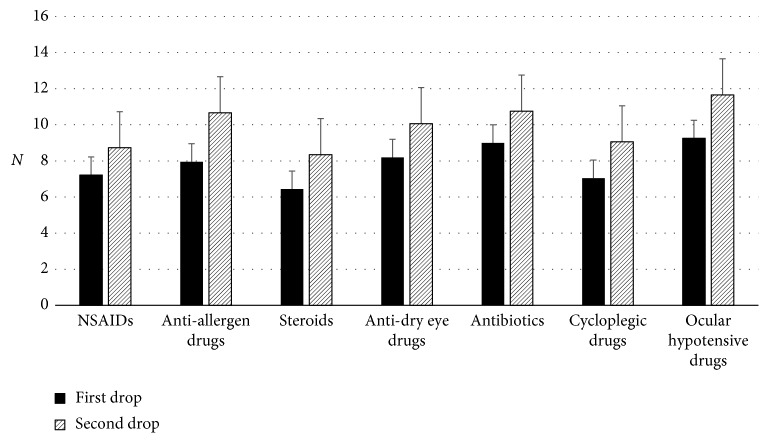
Comparison of squeezing force among categories (*N* = 73). NSAID: nonsteroid anti-inflammatory drugs.

We investigated the relationship with squeezing power by category of ocular disease. A total number of 73 eyedropper bottles, including those containing ocular hypotensive drugs (29 bottles), antidry eye drugs (10 bottles), antibiotics (9 bottles), steroid (8 bottles), antiallergen drugs (7 bottles), cycloplegic drugs (5 bottles), and nonsteroid anti-inflammatory drugs (NSAIDs) (5 bottles), were selected for this analysis because the number of enrolled eyedropper bottles was relatively large in these categories. Squeezing force for the second eye drop was significantly greater than that for the first eye drop in all categories. Both squeezing forces of the first and second drops showed no significant difference among investigated categories (Figure 2).

### 3.5. Effects of Filter Membrane to Squeezing Force

**Figure 3:**
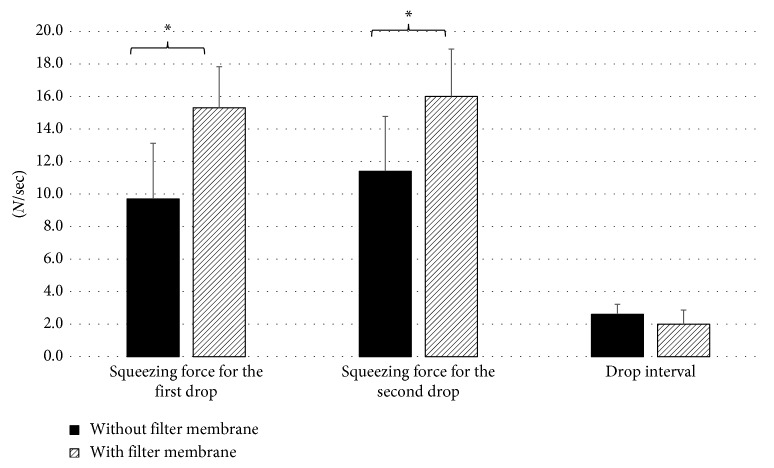
Effects of the filter membrane to squeezing force (*N* = 16). ^*∗*^ *P* < 0.001, the Mann–Whitney *U* test.

The effects of filter membranes to squeezing force were investigated using eight pairs of eyedropper bottles containing the same drug contents. The shape of these eyedroppers was round type. The squeezing forces of the first and second drops were significantly greater than those without filter membranes (*P* < 0.001), but the interval of the first and second drops was not significantly different although eyedropper bottles with filter membranes showed slightly shorter intervals than those without filter membranes (Figure 3).

### 3.6. Multivariate Regression Analysis for Associated Factors

Multivariate regression analysis was performed on factors that affect squeezing force of eyedropper bottles employing all eyedropper bottles. The shape of the eyedropper, generic or brand eye drops, presence of the filter membrane, and drug category were explanatory factors. The round shape of the eyedropper (*P*=0.0001) and the presence of the filter membrane (*P*=0.002) were detected as significant factors to the first eye drop. The shape of eyedropper bottles and the presence of a filter membrane also showed a significant association to the second drop and the interval.

### 3.7. Correlations among Investigated Parameters

**Figure 4:**
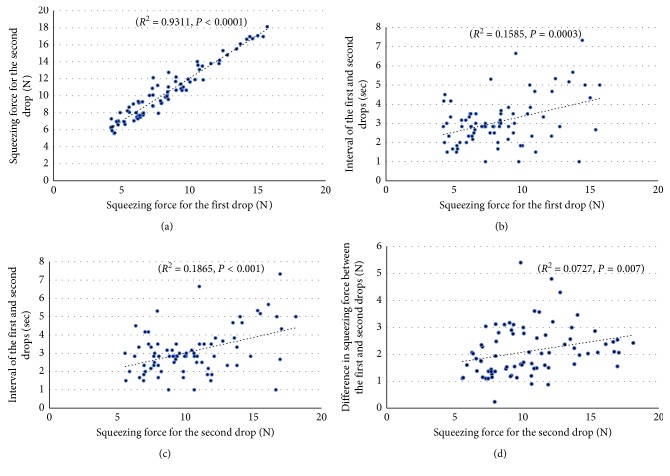
Correlation of investigated parameters (*N* = 78): (a) correlation between squeezing force of the first drop and second drops; (b) correlation between squeezing force of the first drop and interval of two drops; (c) correlation between squeezing force of the second drop and interval of two drops; (d) correlation between squeezing force of the second drop and difference in squeezing force of two drops.

The first drop squeezing force and the second drop squeezing force showed a very strong correlation (*R* ^2^ = 0.9311, *P* < 0.0001) (Figure 4(a)). Those which also had a significant correlation between the investigated parameters are between the first drop squeezing force and the interval (*R* ^2^ = 0.1585, *P*=0.0003) (Figure 4(b)), between the second drop squeezing force and the interval (*R* ^2^ = 0.1865, *P* < 0.0001) (Figure 4(c)), and between the second drop squeezing force and difference in two drops squeezing force (*R* ^2^ = 0.0727, *P*=0.0065) (Figure 4(d)), but these correlations were moderate or weak. The relationship between the interval and the difference in two drops squeezing force showed a related tendency (*P*=0.079). No significant correlation was found between the first drop squeezing force and the difference in two drops squeezing force (Supplemental Figures 1 and 2).

## 4. Discussion

This study compared the squeezing force required for the first and second drops of ophthalmic eye drops and the dispersing time interval between these two drops using commonly employed prescription ophthalmic solutions. There is a difference of more than 3-fold in the squeezing force required for the first drop. The interval between the first drop and the second drop is positively correlated with the squeezing force of the first drop. There is a large difference of more than 20-fold among the eyedropper bottles in the interval between the first and second drops. The interval between two drops is significantly correlated with the squeezing force for the second drop but not for the first drop. The difference in squeezing force was found to be the most influenced by the shape of the eyedropper bottle and the presence of a filter membrane, but not by the category of eye drops, and branded or generic eye drops.

Eyedropper bottles requiring greater squeezing force may instill multiple eye drops in one instillation, and the shapes of eyedroppers and the presence of filter membranes are related to increased squeezing force. The large difference in squeezing force among eyedropper bottles revealed in the current study is consistent with previous studies [8–11, 16]. The variation of squeezing force was four-to six-fold among these previous studies.

Moore et al. reported the pinch strength of glaucoma patients had wide variability. It had been reported that 40 percent of enrolled patients had a maximum pinch strength that was lower than the necessary maximum squeezing force of the investigated eyedropper bottles [8]. Drew and Wolffsohn also reported that some of their glaucoma patients may experience difficulty and uncomfortableness when squeezing eyedropper bottles [10].

The usability decreased along with the increasing squeezing force among eyedropper bottles having the squeezing force of 14.7 N or more [16]. Of 87 eyedropper bottles tested in this study, 10 (11.5%) eyedropper bottles required the same or greater than 14.7 N of a squeezing force. A half of these were eyedropper bottles with filter membranes. Filter membranes are useful to alleviate preservative-related adverse effects, but further modulation improving squeezing force may be necessary. Furthermore, many patients having chronic ocular diseases, including glaucoma, often use multiple types of eyedropper bottles [6]. The differences in squeezing force and interval among eyedropper bottles may hinder proper instillation of eye drops.

The volume of the conjunctival sac is approximately 20 *µ*l, and the one eye drop volume is sufficient to exert the pharmacological effect. Hennessy et al., however, reported that 22.0% of patients instilled two or more than two eye drops in a single attempt, [3] which may result in increasing the possibility of adverse effects and medical expenses. Therefore, eyedropper bottles having greater differences and longer intervals between the first eye drop and the second eye drop are necessary to prevent multiple eye drop instillation in a single attempt. The current study shows the shape of eyedropper bottles significantly related to these points. Eyedropper bottles with center-dimpled shapes showed greater force differences between the first and second drops, which means that this shape is useful for avoiding multidrop instillation. Yoshikawa et al. reported that the dispensing time was mostly influenced by the diameter of the inner aperture of bottles [7]. When we decide on the design of eyedropper bottles, we have to pay attention to these points. Taken together, all eyedropper bottles are required to have similar and lower squeezing force with longer intervals for proper eye drop medication.

Many factors may influence the squeezing force, such as the viscosity of the ophthalmic solution, surface tension of the ophthalmic solution, design of the eyedropper tip, and the shape of the eyedropper bottles [17]. The current study showed the shape of the eyedropper bottles and the presence of filter membranes were significantly associated with squeezing force. Eyedropper bottles with center-dimpled shapes showed significantly lower squeezing force and smaller squeezing force variation than other shapes, regardless of drug categories.

In this study, we examined more than 80 kinds of eyedropper bottles, but because there are more ophthalmic bottles that can be used clinically, we need to verify this result with even more eyedropper bottles. Many factors have been reported to affect squeezing force, such as the viscosity of ophthalmic solutions, the temperature in the testing room, the tilting angle of the eyedropper bottles, and the remaining amount of eye drops in the eyedropper bottles, in addition to the currently investigated factors [9, 11, 16, 17]. Therefore, further investigation is required. We employed multidose bottles only in the current study. Single-dose containers are becoming popular. Since single-dose containers are smaller and likely more difficult to handle, it is necessary to evaluate squeezing force and interval of these containers.

Conner et al. reported that approximately 20% of ophthalmic patients had difficulty squeezing their eye drop bottle [9] We recommend that the future eye drop bottle should satisfy the following conditions: dimple shape bottle, required pinch strengths for the 1^st^ drop and 2^nd^ drop are less than 6N and more than 10 N, and longer than 3 seconds of interval between two drops.

## 5. Conclusions

There were a large variation of the squeezing force and interval of eye drops among eyedropper bottles. The shape of the eyedropper bottle and the presence of the filter membrane may influence squeezing force and eye drop interval. The ease and accurate instillation are very important for proper eye drop treatment, but the great difference in squeezing force and interval among eye drop bottles may result in poor eye drop treatment. We reported that the number of aged subjects with glaucoma will increase in the near future [18]. The older the patient, the more likely there will be physical difficulties in administering eye drops. Proper use of eye drops is fundamental to ophthalmic treatment, and it is therefore necessary to develop eyedropper bottles or eye drop aids focusing on squeezing forces and eye drop interval. Bottle design is important for proper use of topical therapeutics. Unification of eyedropper bottles may be one resolution to standardize eye drop instillation dynamics.

## Figures and Tables

**Table 1:** Investigated eyedropper bottles.

Category	Number	Number of filter membrane
Ocular hypotensive	35	6
Dry eye	11	1
Antibiotics	9	0
Steroid	8	0
Antiallergy	8	1
Nonsteroid anti-inflammatory	4	0
Asthenopia	2	0
Mydriatics	5	0
Topical anesthesia	2	0
Cataract	1	0
Immunosuppressant	1	0
Total	86	8

**Table 2:** Measured values.

	Mean	SD	Lower 95% CI	Upper 95% CI	Max.	Min.	Max./min.
Squeezing force of the first drop (N)	8.3	3.0	7.7	9.0	15.7	4.2	3.7
Squeezing force of the second drop (N)	10.4	3.2	9.7	11.1	18.1	5.6	3.3
Interval (sec)	3.1	1.2	2.8	3.4	7.3	1.0	7.3
Difference in two squeezing forces (N)	2.1	0.8	1.9	2.2	4.8	0.2	20.7

CI: confidential interval.

**Table 3:** Comparison of squeezing force and shape of eyedropper bottles.

	Shape	Mean (N)	SD	Lower 95% CI	Upper 95% CI
First drop	Round	9.2	3.2	8.3	10^*∗*,*∗∗*^
	Dimple	6.7	1.3	5.9	7.5^*∗*,*∗∗*^
	Square	6.5	2.3	5	7.9^*∗∗*^

Second drop	Round	10.9	3.4	10	11.9
	Dimple	9.8	1.6	8.9	10.7
	Square	8.7	2.7	7	10.5

CI: confidential interval; ^*∗*^ *P*=0.02; ^*∗∗*^ *P*=0.01; Tukey–Kramer HSD test.

**Table 4:** Comparison of drop interval and shape of eyedropper bottles.

	Shape	Shape (number)	Mean	SD	Lower 95% CI	Upper 95% CI
First drop	Round	Round (59)	2.9	1.3	2.6	3.3^*∗*,*∗∗*^
	Dimple	Dimple (10)	3.8	0.7	2.4	3.2^*∗*,*∗∗*^
	Square	Square (9)	4	1.2	3.2	4.8^*∗∗*^

Second drop	Round	Round (59)	1.8	0.6	1.6	1.9^¶,¶¶^
	Dimple	Dimple (10)	3.1	0.6	2.7	3.4^¶,¶¶^
	Square	Square (9)	2.3	1.2	1.5	3^¶¶^

CI: confidential interval; ^*∗*^ *P*=0.04; ^*∗∗*^ *P*=0.03; ^¶^ *P*=0.01; ^¶¶^ *P* < 0.001; Tukey–Kramer HSD test.

## Data Availability

The data used to support the findings of this study are available from the corresponding author upon request.

## Supplementary Materials

Supplementary Materials Table A: list on investigated eyedroppers. Supplemental Figure 1: correlation between the second drop squeezing force and the difference in two drops squeezing force. Supplemental Figure 2: correlation between the difference in two drops squeezing force and interval time of two drops.
