# Supplementary material for: Wide Variation of Squeezing Force and Dispensing Time Interval among Eyedropper Bottles
Source: J Ophthalmol. 2019 Apr 16;2019:7250563. doi: 10.1155/2019/7250563 (PMC6501172; doi:10.1155/2019/7250563)
Supplement: Supplementary Materials — Table A: list on investigated eyedroppers. Supplemental Figure 1: correlation between the second drop squeezing force and the difference in two drops squeezing force. Supplemental Figure 2: correlation between the difference in two drops squeezing force and interval time of two drops. [file 7250563.f1.zip › 7250563.f1/Supplemental table.pdf]

Supplemental Table

Serial number of investigated eyedropper	General drug name	blanded or generic drug
1	Latanoprost	bland
2	Latanoprost	generic
3	Latanoprost	generic
4	Latanoprost with filter membrane	generic
5	Timolol	bland
6	Timolol	generic
7	Timolol	generic
8	Timolol	generic
9	Timolol	generic
10	Timolol with filter membrane	generic
11	Carteolol	bland
12	Carteolol	generic
13	Carteolol	generic
14	Carteolol with filter membrane	generic
15	Isopropyl unoprostone	bland
16	Isopropyl unoprostone	generic
17	Isopropyl unoprostone with filter membrane	generic
18	Bunazosin	bland
19	Tafluprost	bland
20	Nipradilol	bland
21	Nipradilol	generic
22	Nipradilol with filter membrane	generic
23	Betaxolol	bland
24	Bimatoprost	bland
25	Ripasudil hydrochloride	bland
26	Brimonidine tartrate	bland
27	Levobunolol	bland
28	Levobunolol with filter membrane	generic
29	Dorzolamide	bland
30	Dorzolamide	generic
31	Dipivefrin Hydrochloride	bland
32	Pilocarpine	bland
33	Latanoprost/timolol maleate fixed combination	bland
34	Tafluprost/timolol maleate fixed combination	bland
35	Carteolol/latanoprost fixed combination	bland
36	Tacrolimus	bland

Supplemental Table (cont'd)

Serial number of investigated eyedropper	General drug name	blanded or generic drug
37	Pirenoxine	bland
38	Hyaluronic acid	bland
39	Hyaluronic acid	generic
40	Hyaluronic acid	generic
41	Hyaluronic acid with filter membrane	generic
42	Diquafosol sodium	bland
43	Sodium hyaluronate	bland
44	Sodium hyaluronate	generic
45	Sodium hyaluronate	generic
46	Sodium hyaluronate	generic
47	Flavin Adenine Dinucleotide/Sodium Chondroitin Sulfate	bland
48	Sodium chondroitin sulfate	bland
49	Oxybuprocaine hydrochloride	bland
50	Fluorometholone	bland
51	Fluorometholone	generic
52	Fluorometholone	generic
53	Betamethasone sodium phosphate/fradiomycin sulfate	bland
54	Betamethasone sodium phosphate	generic
55	Prednisolone	generic
56	Hydrocortisone	generic
57	Dexamethasone	generic
58	Phenylephrine hydrochloride	generic
59	Neostigmine methylsulfate sodium chloride	generic
60	Tropicamide phenylephrine hydrochloride	generic
61	Atropine	generic
62	Tropicamide/phenylephrine	generic
63	Levofloxacin	bland
64	Levofloxacin	generic
65	Levofloxacin	generic
66	Gatifloxacin hydrate	bland
67	Cefmenoxime hydrochloride	bland
68	Chloramphenicol Colistin sodium methanesulfonate	generic
69	Ofloxacin	generic
70	Gentamicin sulfate	generic
71	Tosufloxacin	bland
72	Oxybuprocaine hydrochloride	bland

Supplemental Table (cont'd)

Serial number of investigated eyedropper	General drug name	blanded or generic drug
73	Cyanocobalamin	generic
74	Cyanocobalamin	generic
75	Ketotifen fumarate	generic
76	Sodium cromoglicate	generic
77	Sodium cromoglicate with filter membrane	generic
78	Tramolast	generic
79	Acitazanolast hydrate	bland
80	Levocabastine hydrochloride	bland
81	Epinastine	generic
82	Peramirolast	bland
83	Diclofenac	bland
84	Diclofenac	generic
85	Pranoprofen	generic
86	Sodium gualenate hydrate	generic
