# Supplementary figures and images for: Wide Variation of Squeezing Force and Dispensing Time Interval among Eyedropper Bottles
Source: J Ophthalmol. 2019 Apr 16;2019:7250563. doi: 10.1155/2019/7250563 (PMC6501172; doi:10.1155/2019/7250563)

Supplemental Figure 1

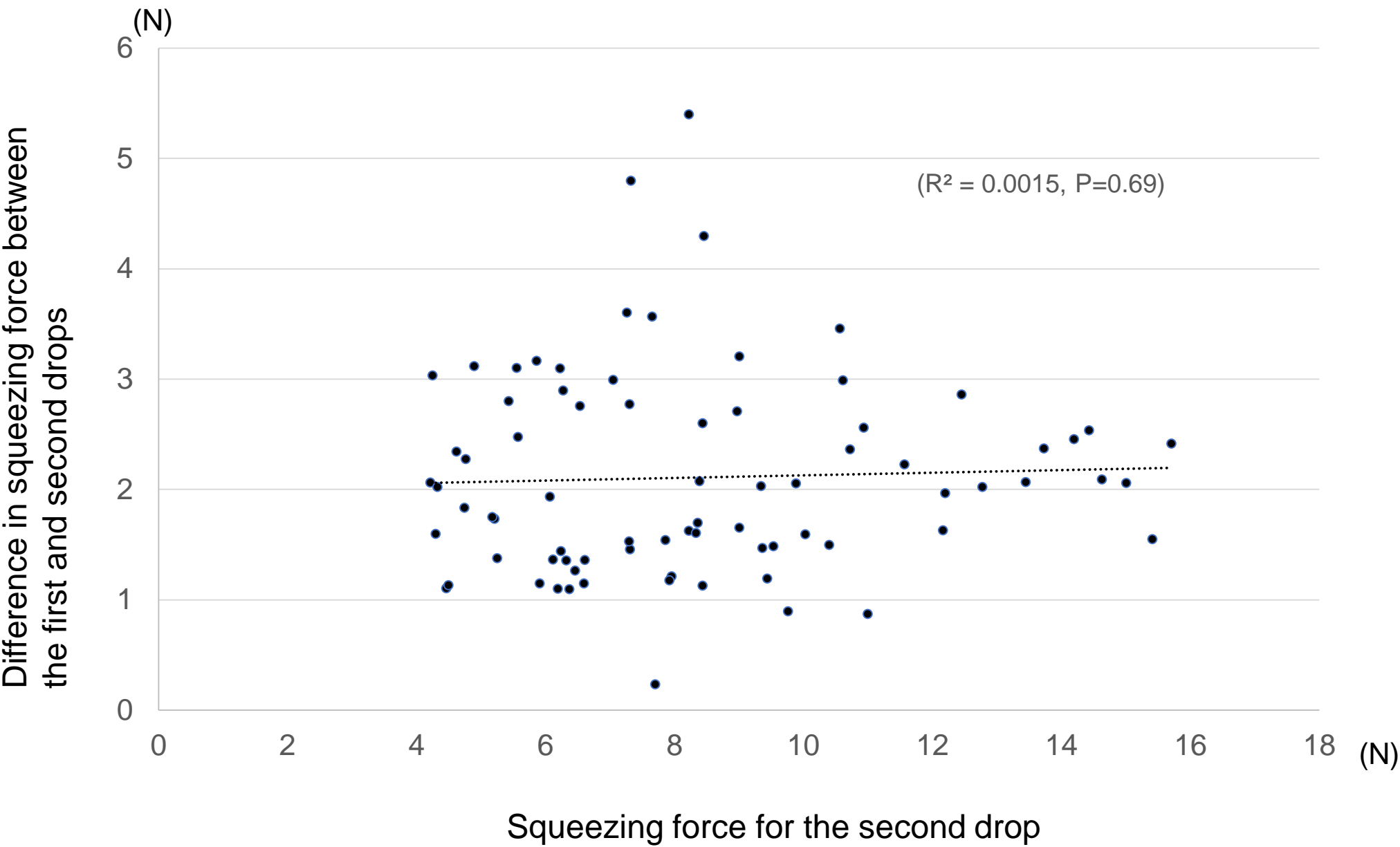

Supplement: Supplementary Materials — Table A: list on investigated eyedroppers. Supplemental Figure 1: correlation between the second drop squeezing force and the difference in two drops squeezing force. Supplemental Figure 2: correlation between the difference in two drops squeezing force and interval time of two drops. [file 7250563.f1.zip › 7250563.f1/supplemental Figure 1.pdf]

Supplemental Figure 2

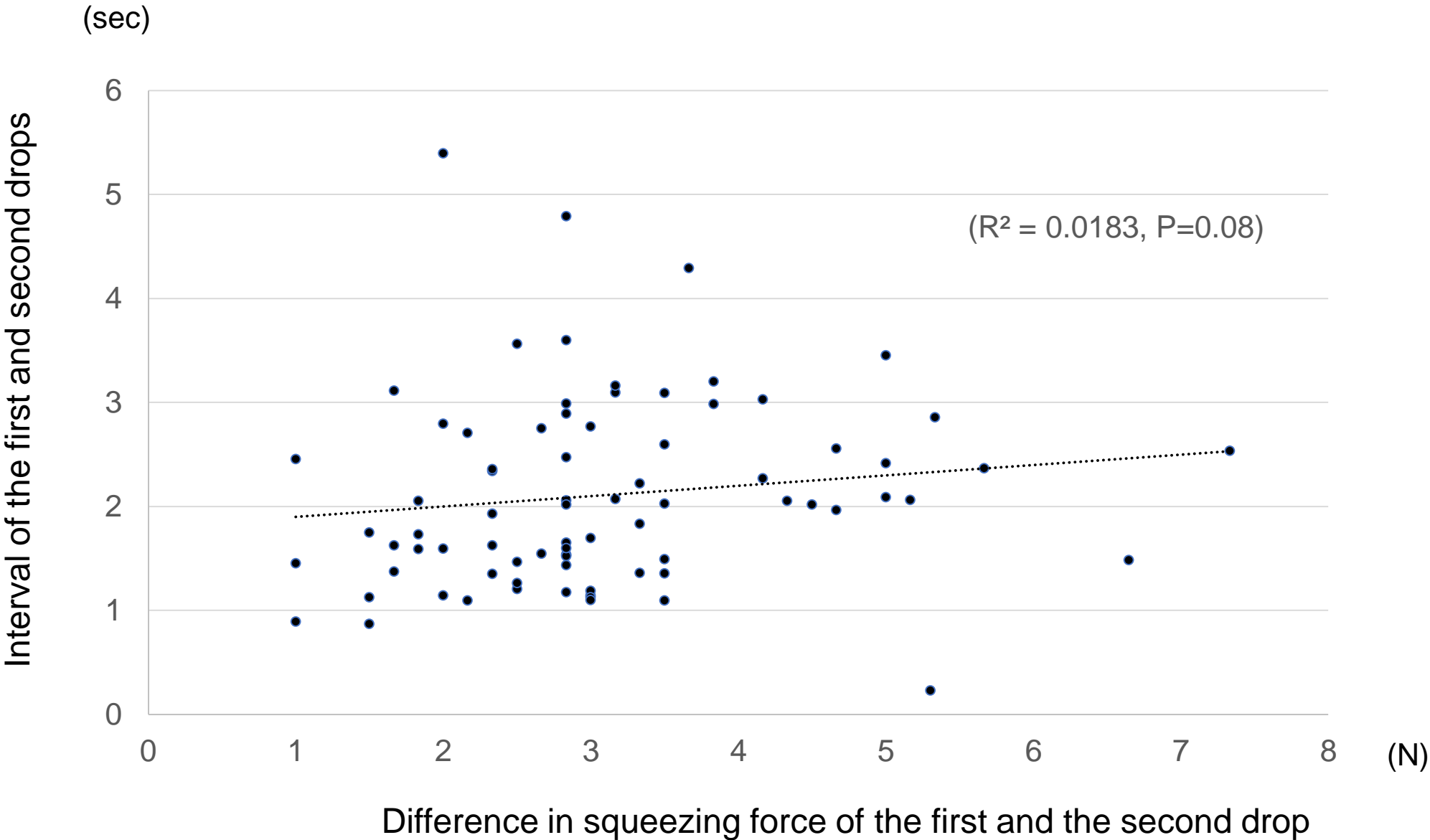

Supplement: Supplementary Materials — Table A: list on investigated eyedroppers. Supplemental Figure 1: correlation between the second drop squeezing force and the difference in two drops squeezing force. Supplemental Figure 2: correlation between the difference in two drops squeezing force and interval time of two drops. [file 7250563.f1.zip › 7250563.f1/supplemental Figure 2.pdf]
